# Belowground bud bank depletion constrains productivity recovery in long established perennial pastures despite nitrogen enrichment

**DOI:** 10.3389/fpls.2026.1910317

**Published:** 2026-09-14

**Authors:** Keyan He, Lili He, Haihong Dang, Jiyun Li, Qian Wang, Qingping Zhou, Jiahao Wang, Xiaoxing Wei

**Affiliations:** 1 Laboratory for Germplasm Research and Utilization on the Qinghai-Tibet Plateau, College of Animal Science and Veterinary Medicine, Qinghai University, Xining, China; 2 College of Grassland Resources, Southwest Minzu University, Chengdu, China; 3 State Key Laboratory of Plateau Ecology and Agriculture in the Headwater Region of the Three Rivers, Qinghai University, Xining, China; 4 Institute of Grassland Research, Chinese Academy of Agricultural Sciences, Hohhot, China

**Keywords:** belowground bud bank, Kentucky bluegrass, nitrogen addition, pasture development, regenerative capacity

## Abstract

Belowground bud banks are the primary source of vegetative regeneration in perennial pastures and may indicate ecosystem regenerative capacity. However, whether bud bank dynamics reflect changes in regeneration potential during pasture development remains unclear. We investigated the responses of belowground bud banks to nitrogen (N) addition in Kentucky bluegrass (*Poa pratensis* L.) pastures established for 4, 7, and 13 years. Three N addition levels, 0 g/m² (control), 5 g/m² (Low-N) and 15 g/m² (High-N), were applied to evaluate changes in belowground bud bank density and its relationships with tiller number and aboveground productivity. Nitrogen addition increased total bud density in 4- and 7-year pastures, with the strongest response under Low-N at year 7. Rhizome bud density declined under High-N addition, whereas tiller bud density remained relatively stable. Total bud density, tiller number, and aboveground biomass peaked at the year 7 and declined markedly by the year 13. No significant relationships were detected between belowground bud density and tiller number or aboveground biomass in the 13-year pasture. These results suggest that belowground bud bank dynamics track changes in tiller recruitment and productivity during pasture development. Their disappearance in long-established pastures suggests a weakening linkage between belowground bud banks, tiller recruitment, and productivity.

## 1 Introduction

Cultivated perennial pastures are vital to livestock production and ecosystem functioning throughout the world (Hopkins and Del Prado, 2007; Zhang, 2021; Ojija, 2024a). Particularly in degraded regions, cultivated perennial pastures are widely established to restore vegetation and ecosystem functions (Gu et al., 2021; Duan et al., 2021; Ojija, 2024b). However, although the productivity of cultivated perennial pastures is generally relatively stable during the early years after establishment, it gradually declines across pasture establishment stages (Li et al., 2022; Dong et al., 2010). Understanding the mechanisms underlying the long-term decline in pasture productivity is therefore important for sustainable pasture management and for maintaining both livestock production and ecological services. The belowground bud bank is considered a primary regenerative structure for maintaining the stable productivity of perennial grass pastures (Ott and Hartnett, 2011; VanderWeide et al., 2014). Compared with the seed bank, the belowground bud bank plays a more important role in vegetation renewal and productivity maintenance in perennial grass pastures (Benson and Hartnett, 2006; Ott et al., 2019). Therefore, changes in the belowground bud bank may provide important insight into the mechanisms of productivity change in cultivated perennial pastures across pasture establishment stages.

Agronomic management practices are commonly used to maintain or increase the productivity of cultivated perennial pastures. Among these practices, fertilization is one of the most important approaches for improving the productive and ecological functions of perennial pastures. Previous studies have shown that short-term phosphorus addition has no significant effect on the belowground bud bank in meadow steppes (Jin et al., 2024), whereas the effects of nitrogen addition on belowground bud density remain controversial. Nitrogen addition has been reported to increase rhizome bud density in some perennial grasses (Wu et al., 2025), such as *Oryza longistaminata* (Shibasaki et al., 2021), but to decrease total belowground bud density in temperate semiarid steppes (Qian et al., 2021). In addition, several studies have suggested that the response of belowground bud density to nitrogen addition depends on nitrogen application level. For example, lower nitrogen addition increased total belowground bud density in *Leymus chinensis*, whereas high nitrogen addition showed no significant effect on total bud density (Fang et al., 2024). Under sufficient nitrogen supply, the increase in tiller bud density of *Leymus chinensis* exceeded that of rhizome bud proliferation (Wang, 2010a). Excessive nitrogen availability may promote preferential nitrogen uptake by roots and suppress rhizome development (Hermans et al., 2006; Gruber et al., 2013), thereby mainly inhibiting rhizome bud formation while exerting relatively limited effects on tiller bud production (Shibasaki et al., 2021). Therefore, it remains unclear whether changes in belowground bud bank density under nitrogen enrichment are associated with variations in tiller recruitment and pasture productivity across different stages of pasture development.

Kentucky bluegrass is an important perennial grass species widely used for establishing cultivated pastures for forage production in America (Dürr et al., 2005; Benson et al., 2023), Europe (Aamlid et al., 2012; McCurdy et al., 2025) and Asia (Zhou et al., 2020). In cold and degraded regions, it is also commonly used for grassland restoration and vegetation recovery because of its strong adaptability and regenerative capacity (Guillard and Inguagiato, 2017; Shaheen et al., 2011; He et al, 2020). Previous studies on productivity of perennial grass pastures have mainly focused on increasing biomass through different cropping patterns (Butkevičienė et al., 2024; Tripolskaja et al., 2025; Meng et al., 2022), maintaining or increasing aboveground biomass through agronomic management practices such as fertilization and mowing (Zhang, 2021; Zhou and Wei, 2023), or enhancing productivity through seed biopriming for stress tolerance (Srivastava et al., 2024). The aboveground biomass of Kentucky bluegrass pasture decreased with the increase of growing years, and significantly decreased at the 5 years after establishment, while it showed no significant difference between the 6 to 9 years after establishment (Wei, 2023). However, it remains unclear how belowground bud bank dynamics contribute to productivity variation during long-term pasture development and whether these responses vary with nitrogen availability and pasture age.

In this study, we investigated how nitrogen addition influences belowground bud bank dynamics and their relationships with tiller recruitment and aboveground productivity in Kentucky bluegrass pastures established for 4, 7, and 13 years. We hypothesized that: (1) belowground bud density would increase under low nitrogen addition but decline under high nitrogen addition; (2) tiller buds and rhizome buds would exhibit contrasting responses to nitrogen addition, with high-N reducing rhizome-bud density while having little effect on tiller-bud density; and (3) the relationships of belowground bud bank density with tiller number and aboveground productivity would vary across pasture establishment stages, and their disappearance in long-established pasture would suggest reduced clonal regenerative capacity.

## 2 Materials and methods

### 2.1 Site description

The field experiment was conducted at the Haibei Experimental Station of Qinghai University (36°59.36’N, 100°52.848’E, elevation 3,156 m), located on the northeastern Qinghai–Tibetan Plateau, China. The site experiences a typical plateau continental climate, characterized by long, cold winters and short, cool summers. The average annual air temperature from 2011 to 2023 was 1.81 °C, and the average maximum and minimum temperature were 22 °C and -20 °C. The average annual precipitation and annual average evaporation were 380 mm and 1650 mm. The Soil in this area is chestnut soil (Chinese Soil Classification System). The soil chemical properties at the 0–30 cm layer was as follows: pH was 8.33, total N was 1.52 g/kg, total P was 1.35 g/kg, soil organic carbon was 20.01g/kg, hydrolysable N was 2.44 mg/kg, Available P was 1.28 mg/kg and Available K was 165.79 mg/kg.

### 2.2 Experimental design and crop management

Kentucky bluegrass pasture was established in 2011 within a 70 m × 40 m field. A 13 m × 51 m fixed experimental area was established within this pasture for the nitrogen addition experiment. To evaluate whether the response of belowground bud banks and aboveground productivity to nitrogen (N) addition changed with pasture establishment stage, a long-term fixed-plot experiment was established in the same pasture field. The same permanent treatment subplots were sampled at three establishment stages: 4 years after establishment in 2014, 7 years after establishment in 2017, and 13 years after establishment in 2023 (**Figure 1**). Within the field, ten replicate blocks were arranged. In each block, three N treatments were randomly assigned to permanent 3 m × 4 m subplots: no N addition control (CK, 0 g/m²), low nitrogen addition (Low-N, 5 g/m²), and high nitrogen addition (High-N, 15 g/m²). The treatment assignment remained unchanged throughout the experiment. Each N treatment had ten independent permanent subplot replicates at each establishment stage.

**Figure 1:**
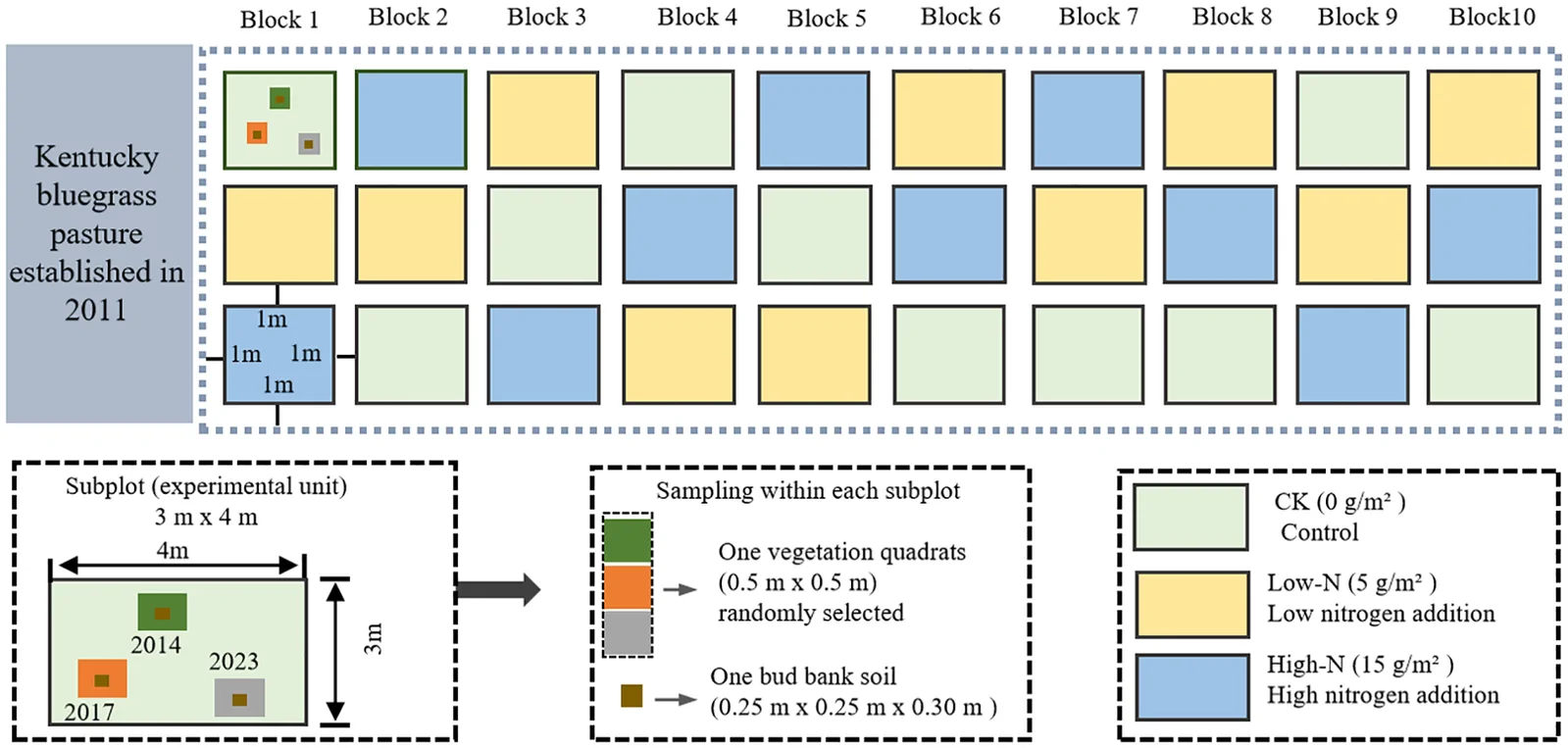
Layout of treatments at experimental block.

Nitrogen fertilizer was applied as urea (CH_4_N_2_O, 46% N) by surface broadcasting during the green-up period of each sampling year. Sampling was conducted in early October at the end of the growing season for each corresponding establishment stage. Previous studies have shown that belowground bud banks remain relatively stable from the end of the growing season to the subsequent green-up period (Teniou et al., 2021).

Because belowground bud bank determination involved destructive sampling of vegetation and soil monoliths, sampling in each year was conducted at previously unsampled positions within each permanent subplot, where vegetation and soil structure remained intact. Previously sampled positions were avoided in subsequent sampling years. In each treatment subplot, one 0.50 m × 0.50 m vegetation quadrat was randomly selected for aboveground biomass harvest at each sampling year. Within each quadrat, one soil monolith of 0.25 m × 0.25 m × 0.30 m was collected to determine belowground bud bank density. This sampling depth was selected because it covers the typical rooting and belowground bud bank distribution depth of Kentucky bluegrass (Wu et al., 2011). Rhizome and tiller buds were separated and classified morphologically following Zhang (2009). Aboveground biomass was clipped at ground level, oven-dried at 105 °C for 30 min to deactivate enzymes, and then dried at 75 °C until constant weight.

Because N treatments were assigned and applied at the subplot level, the permanent subplot was considered the experimental unit. Thus, each N treatment had ten independent subplot-level observations at each establishment stage.

### 2.3 Data analysis

Data on belowground bud density, tiller number, and aboveground biomass were tested for normality using the Shapiro–Wilk test and for homogeneity of variances using Levene’s test. Treatment effects were analyzed using linear mixed-effects models. Establishment stage (4, 7, and 13 years after establishment), N treatment (CK, Low-N, and High-N), and their interaction were treated as fixed effects, whereas block and permanent subplot identity nested within block were included as random effects. Pairwise comparisons were performed using Tukey-adjusted estimated marginal means when significant effects were detected. To examine the relationships among belowground bud density, tiller number, and aboveground biomass, linear regression analyses were conducted and evaluated using 95% confidence intervals. All statistical analyses were performed using R version 4.3.3 (R Foundation for Statistical Computing, Vienna, Austria). Bar charts and regression figures were generated using Origin 2023 (OriginLab Corporation, Northampton, MA, USA). Differences were considered significant at *P* < 0.05.

## 3 Results and analysis

### 3.1 Effects of nitrogen addition and establishment duration on belowground bud bank density

Both nitrogen addition and establishment duration, as well as their interaction, had significant effects on tiller bud density, rhizome bud density and total bud density of Kentucky bluegrass (**Figure 2**). Rhizome bud density and total bud density peaked under Low-N (**Figures 2A, C**), but declined significantly under High-N. In contrast, tiller bud density did not differ significantly between the two nitrogen addition levels (**Figure 2B**). Across the durations, total and rhizome bud density of Kentucky bluegrass reached their highest levels at the 7th year of establishment, whereas both declined markedly by the 13th year (**Figure 2C**). In the 13-year-old pastures, the total bud density was composed almost entirely of tiller buds, with rhizome bud density approaching zero (**Figures 2C**, **3D**).

**Figure 2:**
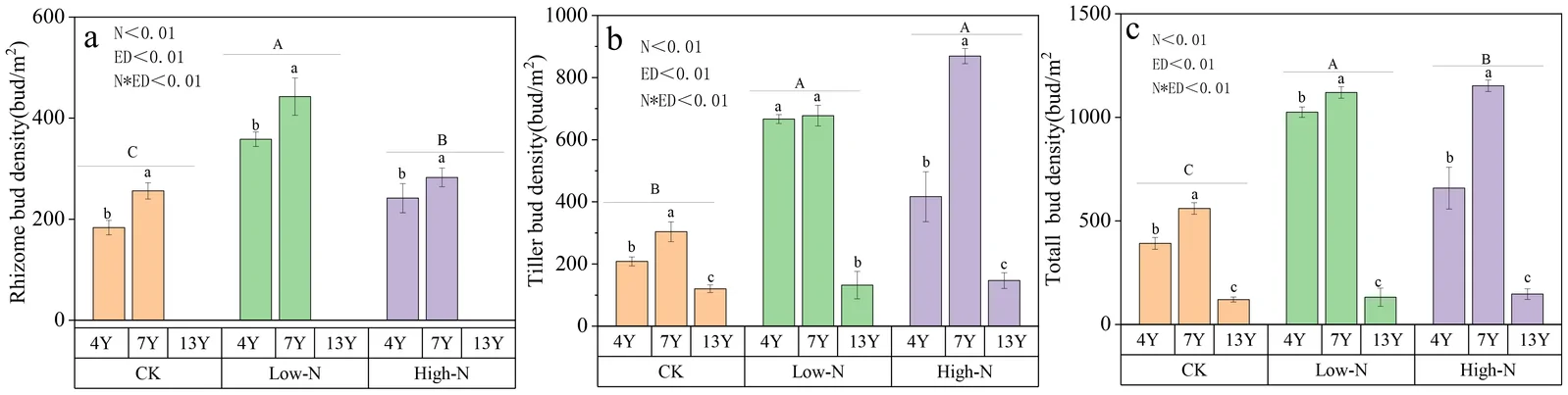
**(a)** Effects of nitrogen addition and establishment duration on rhizome bud density of Kentucky bluegrass; **(b)** Effects of nitrogen addition and establishment duration on tiller bud density of Kentucky bluegrass; **(c)** Effects of nitrogen addition and establishment duration on total bud density of Kentucky bluegrass. Bars represent means ± SE (n = 10). Different lowercase letters indicate significant differences among pasture establishment stages within the same N treatment, whereas different uppercase letters indicate significant differences between N-addition treatments and the control (*P* < 0.05). N, nitrogen addition; ED, establishment duration; N×ED, interaction of nitrogen addition and establishment duration.

**Figure 3:**
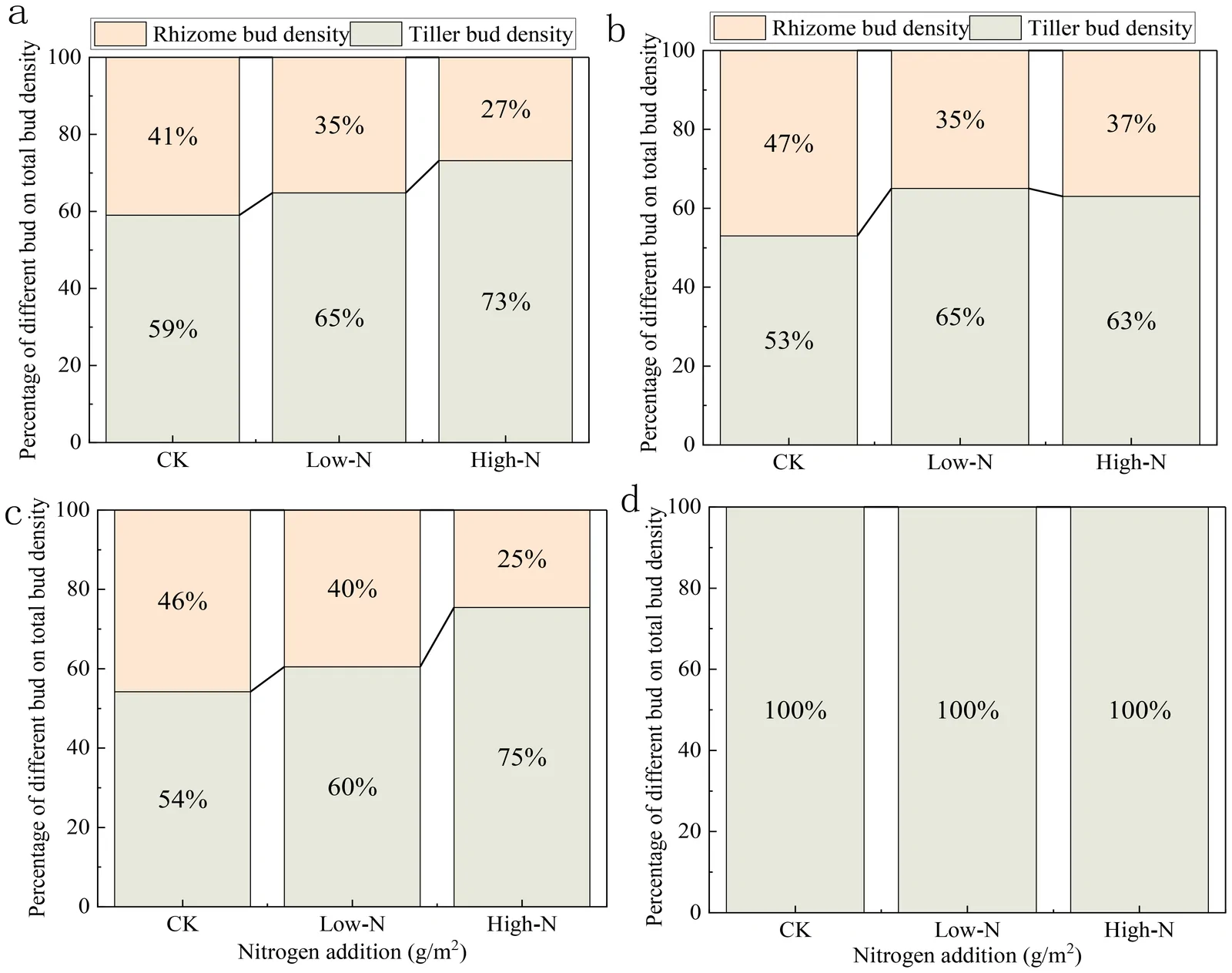
Proportions of tiller and rhizome buds in the total belowground bud bank of Kentucky bluegrass under different nitrogen addition levels across establishment durations. **(A)**, Combined data showing the overall proportion of each bud type across all years; **(B–D)**, Bud composition in 4-, 7-, and 13-year-old pastures, respectively. Values represent the mean of ten replicates. N treatments: Control (no N), Low-N (5 g/m²), and High-N (15 g/m²).

Tiller buds consistently accounted for more than 50% of the total belowground bud density across all establishment durations (4, 7, and 13 years). Nitrogen addition further increased the proportion of tiller buds, with low-N and high-N treatments leading to a 6% and 14% increase, respectively, compared to the unfertilized control (**Figure 3A**). At 4 and 7 years post-establishment, both tiller and rhizome buds contributed substantially to the total bud bank. However, by year 13, rhizome bud density had nearly disappeared, and the bud bank was almost exclusively composed of tiller buds (**Figure 3D**).

### 3.2 Effects of nitrogen addition and establishment duration on tiller production

Nitrogen addition, establishment duration and their interaction had significant effects on both vegetative and total tiller numbers of Kentucky bluegrass (**Figure 4**). Total and vegetative tiller numbers increased significantly with nitrogen input, with the highest values observed under high-N addition (**Figure 4A**). In contrast, reproductive tiller number was primarily influenced by establishment duration and showed no significant response to nitrogen application. It peaked at the 4th year after establishment and declined to its minimum by the 13th year (**Figure 4B**). vegetative tiller number at year 7 was significantly higher than at years 4 and 13 under both nitrogen treatments (**Figure 4C**).

**Figure 4:**
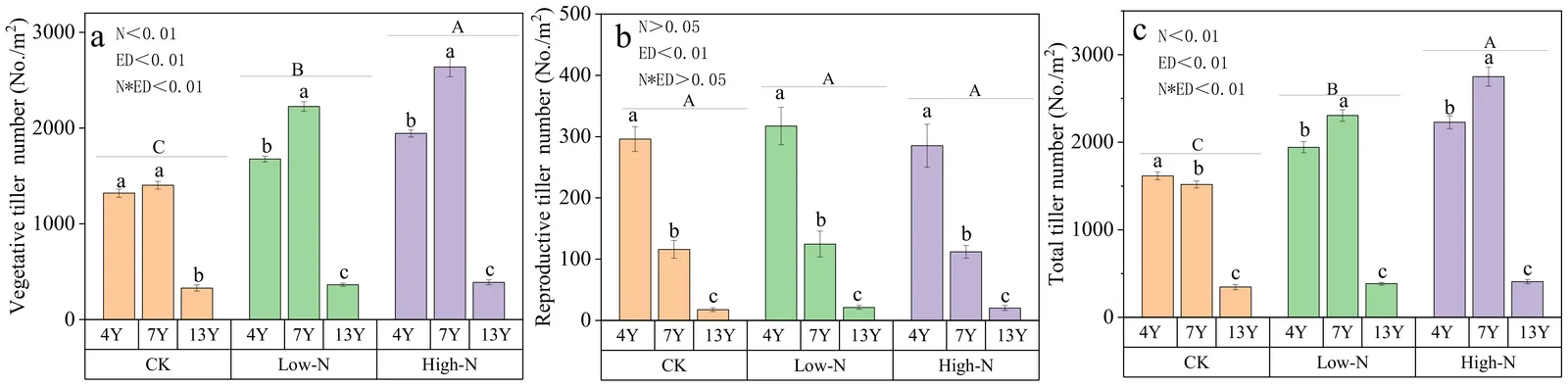
**(a)** Effects of nitrogen addition and establishment duration on vegetative tiller number of Kentucky bluegrass; **(b)** Effects of nitrogen addition and establishment duration on reproductive tiller number of Kentucky bluegrass; **(c)** Effects of nitrogen addition and establishment duration on total tiller number of Kentucky bluegrass. Bars represent mean ± SE (n = 10). Lowercase indicates significant differences among the establishment durations. Capital letter above the bar shows significant differences between nitrogen additions and control. Different lowercase letters indicate significant differences among pasture establishment stages within the same N treatment, whereas different uppercase letters indicate significant differences between N addition treatments and the control (*P* < 0.05). N, nitrogen addition; ED, establishment duration; N×ED, interaction of nitrogen addition and establishment duration.

### 3.3 Effects of nitrogen addition and establishment duration on aboveground biomass

Aboveground biomass of Kentucky bluegrass was significantly influenced by nitrogen addition, establishment duration, and their interaction (**Figure 5**). Aboveground biomass peaked at 7 years after establishment under both nitrogen treatments, and was significantly higher than at 4 and 13 years (*P* < 0.05). In contrast, biomass declined markedly by year 13, indicating a reduction in productivity in long-established pastures despite nitrogen enrichment.

**Figure 5:**
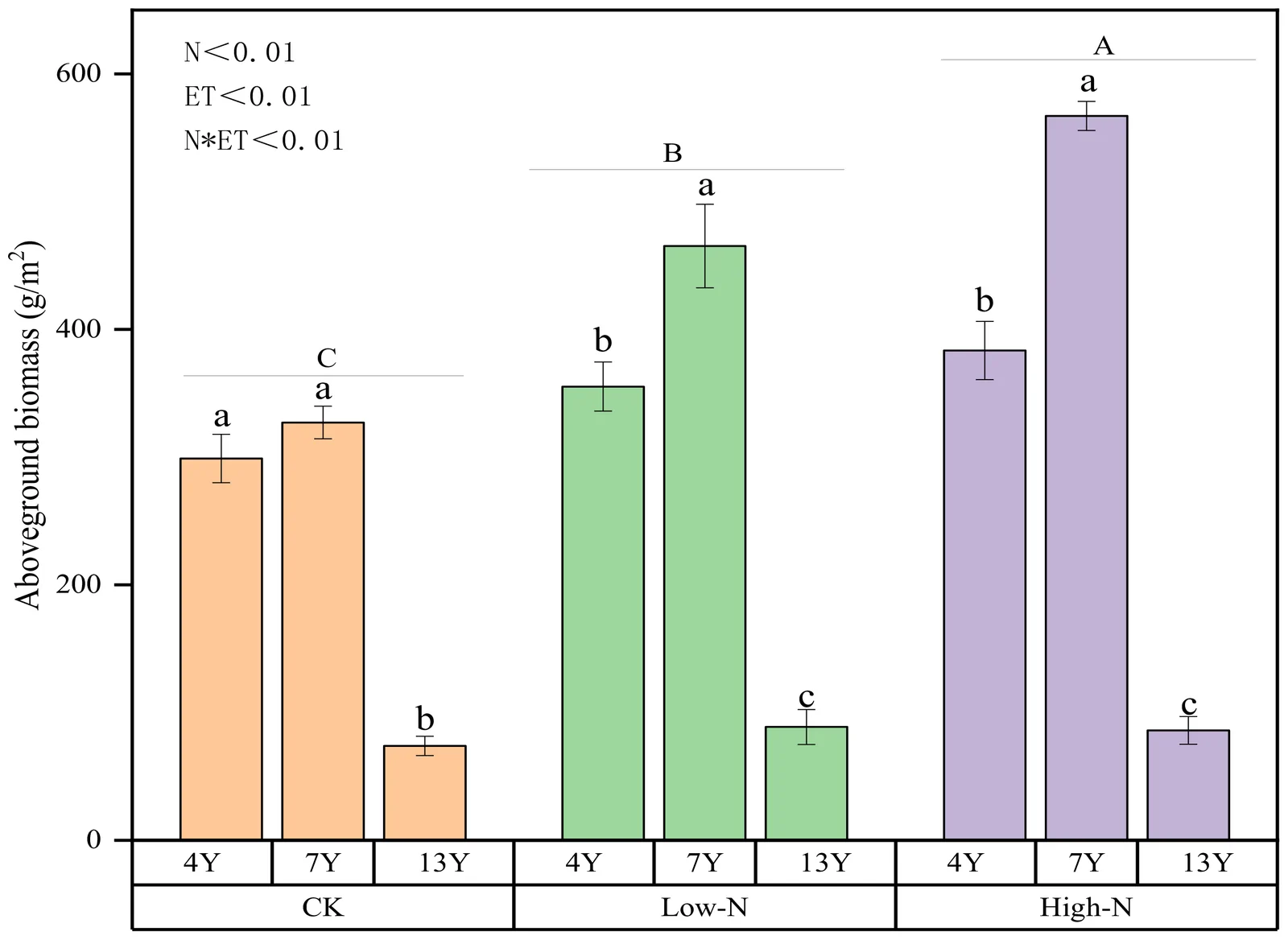
Effects of nitrogen addition and establishment duration on aboveground biomass of Kentucky bluegrass. Bars represent mean ± SE (n = 10). Lowercase indicates significant differences among the establishment time. The capital letter above the bar shows significant differences between nitrogen additions and control. Different lowercase letters indicate significant differences among pasture establishment stages within the same N treatment, whereas different uppercase letters indicate significant differences among the three N treatments (*P* < 0.05). N, nitrogen addition; ED, establishment duration; N×ED, interaction of nitrogen addition and establishment duration.

### 3.4 Relationships of belowground bud density with tiller number and aboveground biomass

When pooling data across all treatments and establishment durations, total belowground bud density was positively correlated with total tiller number (R² = 0.85, *P* < 0.01; **Figure 6A**), and total tiller number was in turn positively correlated with aboveground biomass (R² = 0.97, *P* < 0.01; **Figure 6B**).

**Figure 6:**
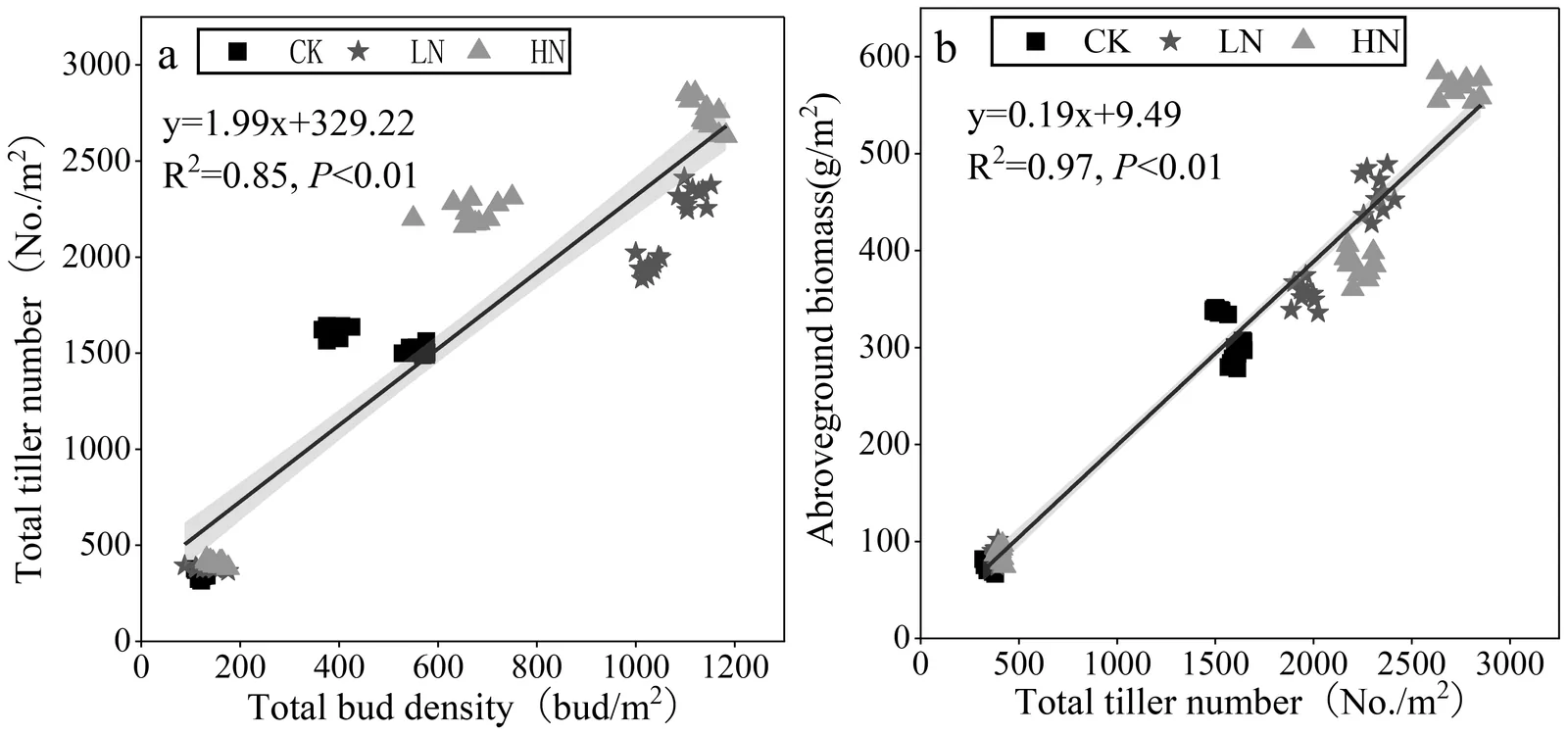
Relationships in Kentucky bluegrass across all treatments. **(A)**, The relationship between the total bud bank of and the number of total tillers; **(B)**, The relationship between the number of total tillers and total biomass. Regression models were fit using the general linear models. Shaded areas represent 95% confidence intervals. Note the equation in the panel “a”, y—total tillers number (No./m^2^), x—total bud density (bud/m^2^). The equation in the panel “b”, y—aboveground biomass (g/m^2^), x—total tiller number (bud/m^2^).

In Kentucky bluegrass pastures established for 4 and 7 years, significant positive linear relationships were observed between belowground bud density and total tiller number under High-N (R² = 0.72, *P* < 0.01, **Figure 7C**; R² = 0.96, *P* < 0.01, **Figure 8C**). Furthermore, under Low-N, total tiller number showed a significant positive correlation with aboveground biomass at both 4 and 7 years (R² = 0.90, *P* < 0.01, **Figure 7E**; R² = 0.79, *P* < 0.01, **Figure 8E**). No significant correlation was found between the aboveground biomass and the total tiller number under High-N condition (**Figure 7F**, 8F).

**Figure 7:**
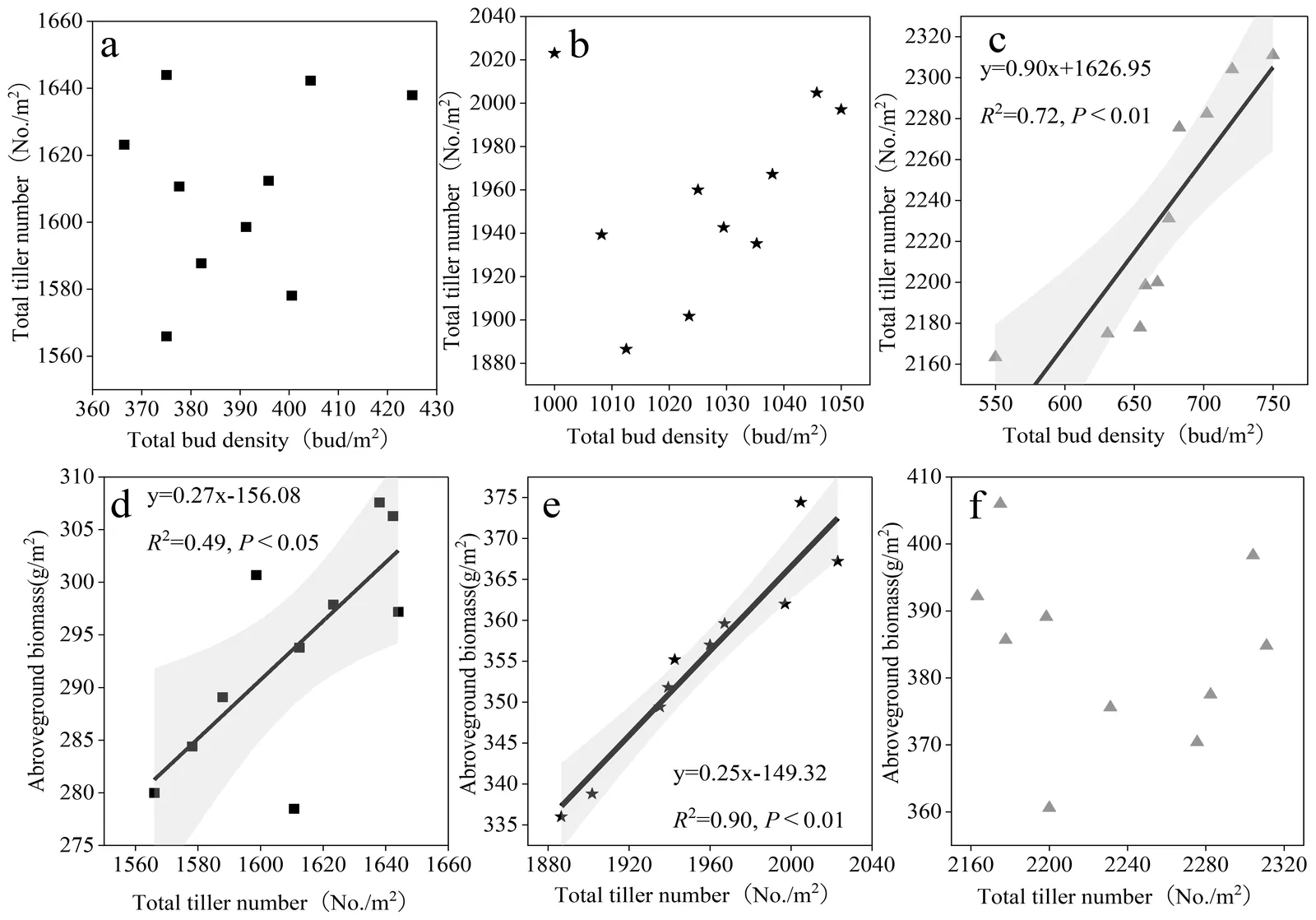
**(a)** Relationship between total belowground bud density and total tiller number in 4-year-old Kentucky bluegrass pastures under the CK; **(b)** relationship between total belowground bud density and total tiller number under the Low-N; **(c)** relationship between total belowground bud density and total tiller number under the High-N; **(d)** Relationship between total tiller number and aboveground biomass under the CK; **(e)** relationship between total tiller number and aboveground biomass under the Low-N; and **(f)** relationship between total tiller number and aboveground biomass under the High-N. Linear regressions were fit separately for each N level. Shaded areas represent 95% confidence intervals.

**Figure 8:**
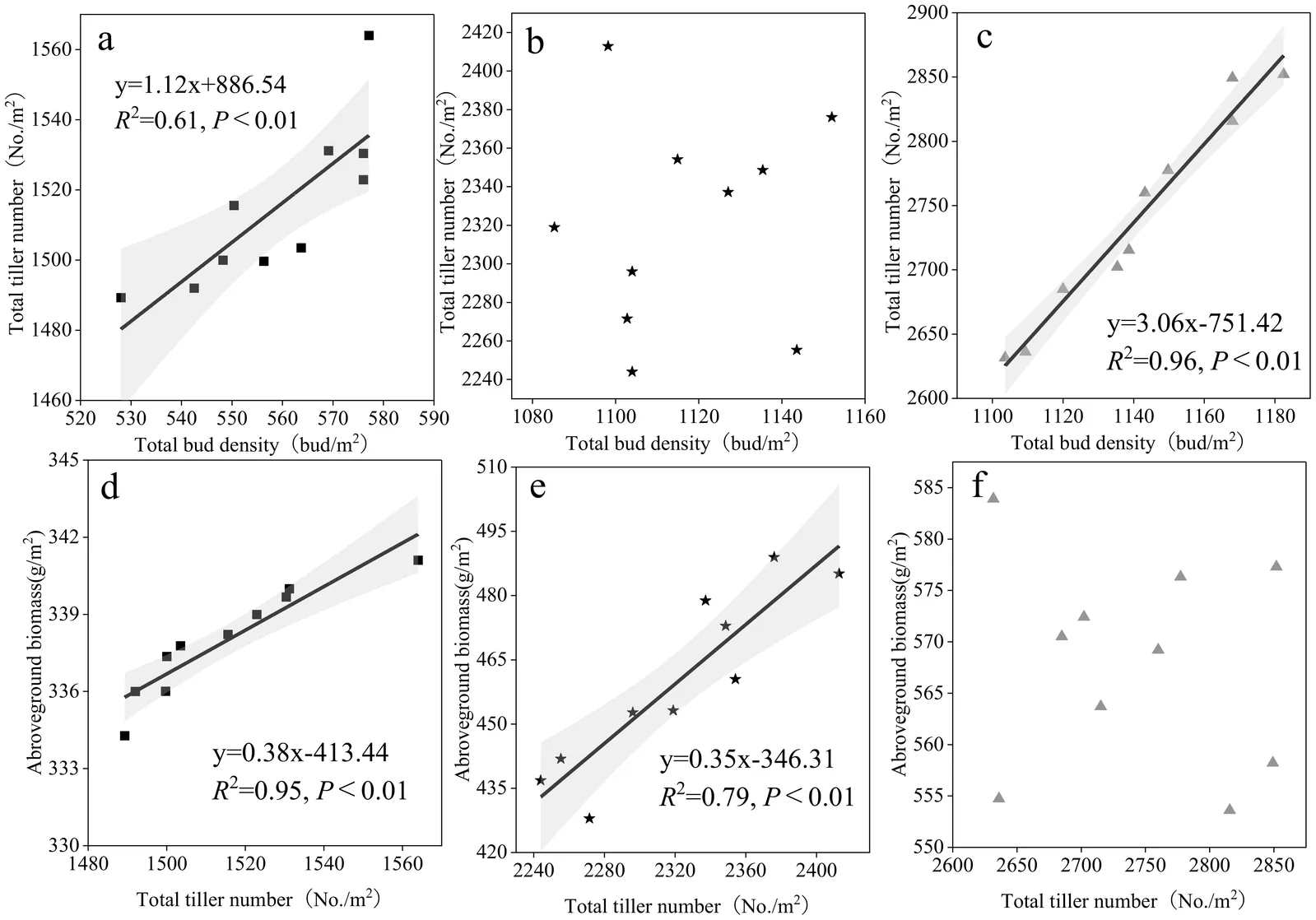
**(a)** Relationship between total belowground bud density and total tiller number in 7-year-old Kentucky bluegrass pastures under the CK; **(b)** relationship between total belowground bud density and total tiller number under the Low-N; **(c)** relationship between total belowground bud density and total tiller number under the High-N; **(d)** Relationship between total tiller number and aboveground biomass under the CK; **(e)** relationship between total tiller number and aboveground biomass under the Low-N; and **(f)** relationship between total tiller number and aboveground biomass under the High-N. Linear regressions were fit separately for each N level. Shaded areas represent 95% confidence intervals.

In the 13-year-old pastures, no significant correlation was found between belowground bud density and tiller number, nor between tiller number and aboveground biomass (**Figure 9**). These results indicate that the contribution of the bud bank to productivity diminishes over time, particularly in aging monocultures, and may serve as a sensitive indicator of declining regenerative capacity and pasture functionality. These results suggest that the contribution of the bud bank to productivity may diminish over time, particularly in aging monocultures, and that changes in this relationship may serve as a sensitive indicator of declining regenerative capacity and pasture functionality.

**Figure 9:**
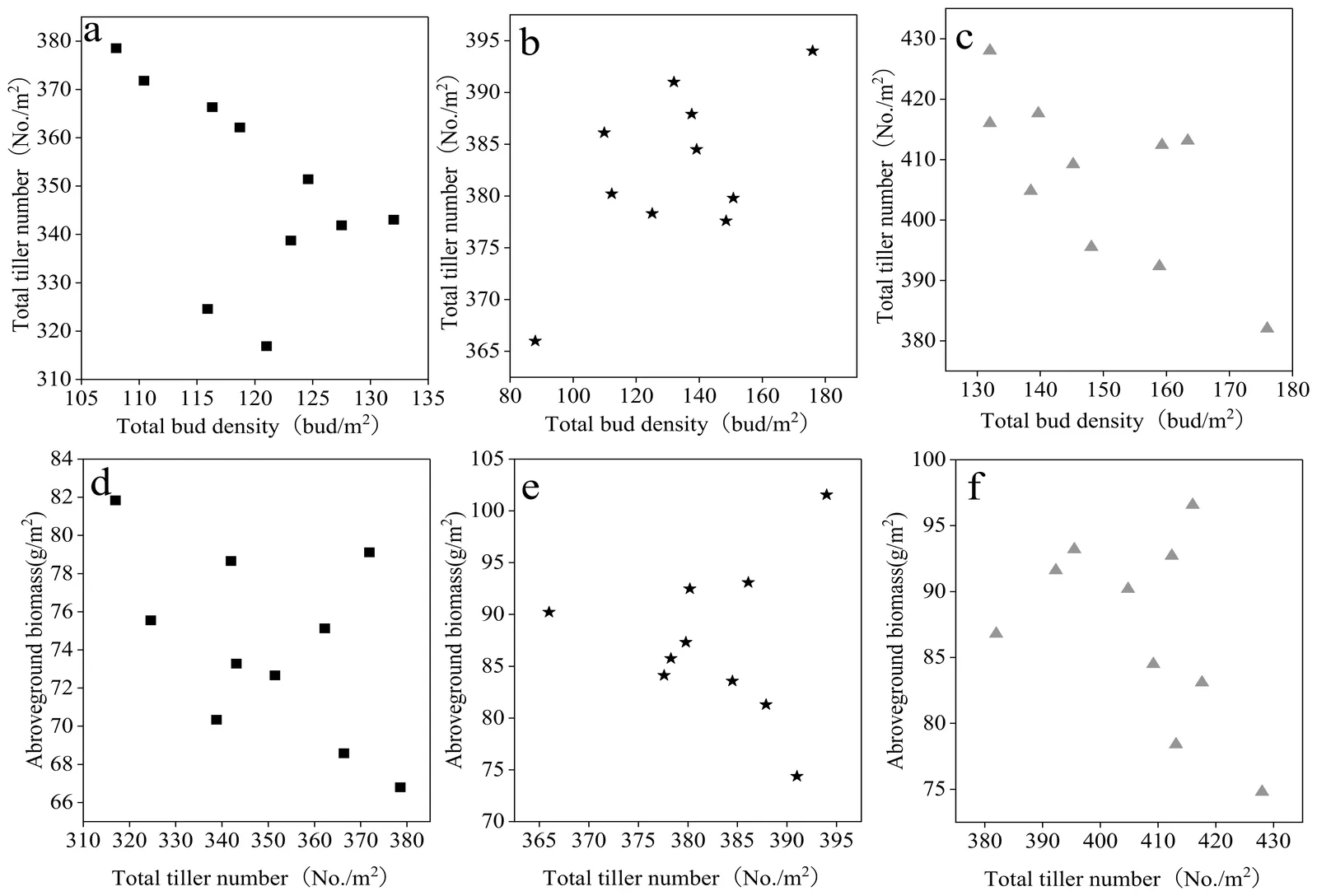
**(a)** Relationship between total belowground bud density and total tiller number in 13-year-old Kentucky bluegrass pastures under the CK; **(b)** relationship between total belowground bud density and total tiller number under the Low-N; **(c)** relationship between total belowground bud density and total tiller number under the High-N; **(d)** Relationship between total tiller number and aboveground biomass under the CK; **(e)** relationship between total tiller number and aboveground biomass under the Low-N; and **(f)** relationship between total tiller number and aboveground biomass under the High-N. No significant linear relationship was observed.

## 4 Discussion

### 4.1 Nitrogen mediated changes in belowground bud banks and clonal regeneration strategies

This study showed that N addition altered the belowground bud bank of Kentucky bluegrass, with the strongest positive response generally observed under Low-N addition (5 g/m²). This finding supports our first hypothesis and is consistent with previous studies showing that N availability can regulate the dormancy, activation, and outgrowth of clonal buds (Tomlinson and Connor, 2004; Ott et al., 2019). Mechanistically, N availability may influence cytokinin synthesis and transport (Takei et al., 2001), thereby promoting cell division and lateral bud sprouting (Bangerth et al., 2000) and enhancing tillering capacity (Rong et al., 2022). Similar positive responses of belowground bud banks to moderate nutrient addition have been reported in perennial grasses such as *Koeleria macrantha*, *Sporobolus heterolepis*, and *Leymus chinensis* (Dalgleish et al., 2008; Yu et al., 2022). However, the response of bud density to N addition was not linear. High-N addition (15 g/m²) did not further increase bud density and, in some cases, reduced total bud density, consistent with previous study reporting suppression of clonal bud activity under nutrient oversupply (Zang et al., 2019). This pattern suggests that excessive N input may shift plant carbon allocation toward aboveground growth at the expense of belowground regenerative organs (Hermans et al., 2006). Therefore, the belowground bud bank may serve as a sensitive indicator of the nutrient threshold beyond which fertilization no longer enhances, and may even impair, the regenerative capacity of Kentucky bluegrass pastures.

The contrasting responses of rhizome buds and tiller buds further indicate that N addition may alter the clonal propagation strategy of Kentucky bluegrass. Rhizome bud density was lower under High-N than under Low-N conditions, whereas tiller bud density was less sensitive to high N input. This result supports our second hypothesis and suggests that tiller buds may be more tolerant of N enrichment than rhizome buds. Several mechanisms may explain this pattern. First, compared with moderate N supply, excessive N availability may shift plant resource allocation toward rapid aboveground growth, reducing investment in rhizomes and other belowground structures involved in clonal spread and resource storage (Poorter et al., 2012; Yan et al., 2019). As a result, rhizome growth and rhizome bud formation may be suppressed under High-N conditions. Second, rhizome growth and rhizome bud development depend heavily on stored carbohydrate reserves (Ott et al., 2019; Fidelis et al., 2014). Reduced carbon investment in storage organs under high N availability may therefore disproportionately constrain rhizome development, contributing to the observed decline in rhizome-bud density. Third, tiller buds and rhizome buds differ in their ecological functions. Tiller buds primarily contribute to rapid local vegetative expansion, whereas rhizome buds are associated with resource storage, long-term persistence, and spatial colonization (Klimesova et al., 2018). Under nutrient rich conditions, maintaining a greater proportion of tiller buds may allow Kentucky bluegrass to maximize short term population expansion, whereas investment in the more conservative rhizome-bud strategy becomes less advantageous. This functional shift may explain the greater tolerance of tiller buds to N enrichment and the decline of rhizome buds under High-N input. Given the contrasting ecological functions of rhizome and tiller buds, shifts in their relative abundance suggest that nitrogen enrichment can alter the balance between persistence-oriented and expansion-oriented clonal growth strategies.

### 4.2 Bud bank dynamics reflect changes in regenerative capacity during pasture development

The variation in the relationships of belowground bud bank density with tiller recruitment and aboveground productivity across pasture establishment stages generally supported our third hypothesis. Significant positive relationships were observed in younger pastures but disappeared at the 13-year stage, suggesting that the contribution of belowground bud banks to tiller recruitment and productivity weakened during long-term pasture development. Both rhizome- and tiller-bud densities tended to peak at the 7-year establishment stage and declined markedly by the 13-year stage. This pattern is consistent with the idea that clonal bud banks may follow a unimodal trajectory during stand development, with an initial expansion phase followed by reduced regenerative capacity in older stands (Klimeš et al., 1997; Ott and Hartnett, 2015). The decline in bud density at the 13-year stage may reflect multiple interacting processes, including nutrient depletion associated with long-term monoculture (Aerts and Chapin, 2000) and reduced meristematic activity resulting from clonal aging (Roach and Smith, 2020). The inability of N addition to restore bud density or productivity at the 13-year stage suggests that productivity decline in older Kentucky bluegrass pastures may not be driven solely by N limitation. Instead, declining bud bank size and function may represent an increasingly important constraint on regeneration and productivity in aging monoculture pastures. Interannual climatic and environmental variability may also influence plant growth and belowground bud bank dynamics. The present study focused on temporal changes in bud bank dynamics and productivity across pasture establishment stages, while future studies incorporating detailed climatic and environmental variables will further examine their potential effects on these long term patterns.

N addition significantly increased vegetative tiller number but had little effect on reproductive tiller number. Reproductive tiller number declined progressively with establishment stage, indicating that reproductive output was more strongly associated with pasture aging than with short term N availability. Similar patterns have been observed in other rhizomatous perennial grasses, such as *Leymus chinensis*, in which heading rate and ear number were more dependent on stand age than on N fertilization (Wang et al., 2010b). Perennial rhizomatous grasses possess both clonal and sexual reproductive pathways (Lv, 2010), and nitrogen enrichment may alter resource allocation between these two reproductive strategies (Yang and Kim, 2016). Under favorable nutrient conditions, plants often invest more resources in clonal propagation because it provides a rapid and efficient means of population expansion (López-Almansa et al., 2003). Consequently, N addition may preferentially stimulate vegetative growth while contributing little to sexual reproduction. These results suggest that N enrichment mainly promotes short term vegetative propagation rather than sexual reproduction in Kentucky bluegrass pasture. The age related decline in reproductive tillers further indicates that long-established pasture may increasingly rely on clonal propagation for population maintenance. Changes in the balance between vegetative and reproductive tillers may therefore provide additional insight into shifts in regeneration strategy during long-term pasture development.

### 4.3 Weakening contributions of bud banks to tiller recruitment and productivity in aging pasture

Our results showed that the relationships of belowground bud density with tiller number and aboveground biomass were jointly influenced by nitrogen addition level and pasture establishment stage. Under high-N conditions, belowground bud density was positively correlated with total tiller number at the 4- and 7-year establishment stages (*P* < 0.05), indicating that elevated nitrogen availability promoted the recruitment of tillers from belowground buds. This finding is consistent with the established role of nutrient availability in regulating bud activation and shoot emergence (Marschner, 2012; Epstein and Bloom, 2005; Tomlinson and Connor, 2004). However, no significant relationship was observed between belowground bud density and aboveground biomass under Ligh-N input, suggesting that increased tiller recruitment did not necessarily translate into greater biomass accumulation. This indicates that, under high nitrogen availability, belowground bud banks primarily contribute to tiller recruitment rather than directly determining aboveground productivity. In contrast, under CK and Low-N conditions, belowground bud density was significantly associated with aboveground biomass, whereas its relationship with tiller number was weak. This pattern may reflect stronger resource limitation under low nitrogen availability. Reduced nutrient supply can constrain photosynthetic capacity (Marschner, 2012; Chen et al., 2022) and promote greater allocation of resources belowground (Poorter and Nagel, 2000), thereby limiting tiller recruitment (Stevens et al., 2004). These findings suggest that the role of belowground bud banks varies with nitrogen availability, being more closely associated with tiller recruitment under high-N conditions but with aboveground productivity under CK and low-N conditions. At the 13-year establishment stage, the relationships between belowground bud density and tiller number and between tiller number and aboveground biomass were no longer evident. This decoupling suggests that belowground bud density may no longer closely track tiller recruitment, and that tiller recruitment may no longer closely track aboveground productivity in long-established pastures, potentially reflecting changes associated with long-term pasture development. One possible explanation is the gradual aging of clonal structures (Thomas, 2013). As perennial clonal populations age, the capacity of belowground buds to generate new tillers and support aboveground growth may decline (Ott et al., 2019). Thus, the stage dependent relationships among belowground bud density, tiller recruitment, and aboveground biomass suggest that the linkage between belowground bud banks and aboveground performance becomes increasingly context dependent during long term pasture development. Together, the decline in bud bank size and the altered coupling between bud bank dynamics and aboveground performance may be associated with reduced regenerative capacity in long-established Kentucky bluegrass pastures, even under continued nitrogen enrichment.

## 5 Conclusion

This study demonstrated that belowground bud bank dynamics in Kentucky bluegrass pasture are jointly regulated by nitrogen addition and pasture establishment stage. Low-N addition (5 g/m²) promoted bud bank development, whereas High-N addition (15 g/m²) reduced rhizome-bud density, indicating that excessive nitrogen input may alter clonal regeneration strategy. Both bud density and vegetative tiller number reached their highest values at the 7-year establishment stage but declined by the 13-year stage, indicating a progressive reduction in clonal regenerative capacity during long-term pasture development. The relationships among belowground bud density, tiller recruitment, and aboveground productivity varied with nitrogen availability and became weaker at the 13-year establishment stage. This pattern suggests that the contribution of belowground bud banks to tiller recruitment and productivity changes during pasture development. Furthermore, nitrogen addition failed to restore either bud bank size or aboveground productivity at the 13-year stage, indicating that productivity decline in long-established pasture cannot be explained by nitrogen limitation alone. Overall, our results highlight the importance of belowground bud banks in regulating regeneration and productivity in perennial pasture. Understanding changes in bud bank dynamics and their contribution to tiller recruitment and productivity may help optimize nitrogen management and pasture renovation strategies.

## Data Availability

The raw data supporting the conclusions of this article will be made available by the authors, without undue reservation.
